# First Detection of Honeybee Pathogenic Viruses in Butterflies

**DOI:** 10.3390/insects13100925

**Published:** 2022-10-12

**Authors:** Metka Pislak Ocepek, Gordana Glavan, Rudi Verovnik, Laura Šimenc, Ivan Toplak

**Affiliations:** 1 Institute of Pathology, Wild Animals, Fish and Bees, Veterinary Faculty, University of Ljubljana, Gerbičeva 60, 1000 Ljubljana, Slovenia; 2 Department of Biology, Biotechnical Faculty, University of Ljubljana, Jamnikarjeva 101, 1000 Ljubljana, Slovenia; 3 Institute of Microbiology and Parasitology, Veterinary Faculty, University of Ljubljana, Gerbičeva 60, 1000 Ljubljana, Slovenia

**Keywords:** butterflies, honeybees, viruses, pathogens transmission

## Abstract

**Simple Summary:**

A decline in the number and diversity of pollinators has been observed worldwide. Various butterfly species are also threatened by extinction. One of the less studied reasons for the pollinator decline are pathogens, although it is known that they can be transmitted between cultivated and some wild pollinator species. The objective of our study was to determine whether pathogenic honeybee viruses can be transmitted to butterflies. Butterfly and bee samples from four locations in Slovenia were analyzed using the molecular method RT-PCR. We considered six bee viruses and found very low levels of four of them, i.e., acute bee paralysis virus (ABPV), black queen cell virus (BQCV), Lake Sinai virus 3 (LSV3), and Sacbrood bee virus (SBV), in the butterfly samples compared to the bee samples, although these viruses were present in large amounts in honeybees from the same locations. The results of our study indicate that butterflies are probably contaminated with bee viruses when they visit the same flowers as honeybees. Therefore, based on the very low viral load, we believe that butterflies are unlikely to be threatened by bee viruses and that they are not important as vectors of viral infection to other pollinators.

**Abstract:**

Several pathogens are important causes of the observed pollinator decline, some of which could be transmitted between different pollinator species. To determine whether honeybee viruses can be transmitted to butterflies, a total of 120 butterflies were sampled at four locations in Slovenia. At each location, butterflies from three families (Pieridae, Nymphalidae, Hesperiidae/Lycenidae) and Carniolan honeybees (*Apis mellifera carnica*) were collected. The RNA of six honeybee viruses, i.e., acute bee paralysis virus (ABPV), black queen cell virus (BQCV), chronic bee paralysis virus (CBPV), deformed wing virus A (DWV-A), Sacbrood bee virus (SBV), and Lake Sinai virus 3 (LSV3), was detected by a specific quantitative method (RT-PCR). The presence of ABPV, BQCV, LSV3, and SBV was detected in both butterflies and honeybees. All butterfly and bee samples were negative for CBPV, while DWV-A was detected only in honeybees. The viral load in the positive butterfly samples was much lower than in the positive bee samples, which could indicate that butterflies are passive carriers of bee viruses. The percentage of positive butterfly samples was higher when the butterflies were collected at sampling sites with a higher density of apiaries. Therefore, we believe that infected bees are a necessary condition for the presence of viruses in cohabiting butterflies. This is the first study on the presence of pathogenic bee viruses in butterflies.

## 1. Introduction

Flower-visiting insects are essential to the existence of natural and agricultural ecosystems because of their pollination function and also play an important role in the food web. Insect diversity, especially of pollinators, is also necessary for human food safety [1,2,3,4]. One of the most studied groups of insects are butterflies [5], which are popular with the public because of their beauty and visibility. Butterflies enjoy great attention from the point of view of species conservation and are considered a model for studies on insect decline because they are highly sensitive to changes in their habitat [5,6]. However, butterflies also have an important scientific value because of the long history of butterfly studies, including their monitoring, which provide a unique source of data for scientific research [7]. The pollination function of butterflies and moths is less pronounced compared to that of other pollinators, but there are plants and regions that can only be pollinated by butterflies, such as flowers with deep tubes or flowers that open only at night [8,9]. Moths are the largest group of nocturnal pollinators and are therefore important for maintaining plant diversity, though they are also pollinators of crops such as flax, herbs, and clover [10,11,12,13].

There is clear evidence that butterflies are declining throughout Europe and other parts of the world [5,7,14,15]. The factors leading to the decline of butterflies are numerous. The most important is habitat destruction and/or degradation due to intensive agriculture and urbanization resulting in habitat loss or fragmentation, which particularly affects habitat-specialized butterfly species [15,16,17]. In addition, the decline of butterflies is also influenced by the excessive use of pesticides and nitrogen in fertilizers [5,18,19,20]. Climate change, while favoring the poleward range expansion of more mobile generalist species [21], is also causing a decline in montane butterflies adapted to colder environments or leading to asynchrony in host plant and butterfly phenology and shifts in distribution [5,22,23].

In addition to environmental changes, predators and pathogens also affect insect survival. Despite the diversity of Lepidoptera and their numerous interactions with other organisms, the effects of pathogens in butterflies and moths have been scarcely studied. In fact, this interaction has been studied primarily in the context of biological control—the use of pathogens to regulate caterpillar populations [24]. However, several pathogens have been identified in butterflies, including fungi, bacteria, protozoa, and viruses [25]. Somewhat more attention has been paid to the protozoan parasite *Ophryocystis elektroscirrha*, which infects the monarch (*Danaus plexippus*), a known migratory butterfly from North America [26,27,28]. In general, pathology in butterflies is not easily observed or diagnosed and requires laboratory testing [25]. Fungi are the most common pathogens in butterflies and some of them can cause high mortality in caterpillars [25,29]. The entomopathogenic fungus *Beauvaria bassiana* has been successfully used for the biological control of the caterpillars of the large white butterfly (*Pieris brassicae*) [30]. In the study of bacterial contamination of monarch species, the bacteria *Pseudomonas aeruginosa* and *Bacillus thuringiensis* were isolated [31,32]; *B. thuringiensis* is the most common commercially produced bacterium for the control of Lepidoptera larvae [33]. Among the common microbial pathogens infecting insects, including butterflies, are intracellular bacteria of the genus *Wolbachia* [34,35]. These bacteria do not kill the host directly but manipulate the host biology in several ways, including inducing reproductive manipulations such as feminization, parthenogenesis, male killing, and sperm–egg incompatibility [36]. Known viruses in butterfly and moth larvae include nuclear polyhedrosis viruses (NPV), which are lethal to the caterpillars, and after death, millions of virus particles are left on the plant to infect new hosts [25]. In addition to NPVs, baculoviruses and cytoplasmic polyhedrosis viruses have also been identified in Lepidoptera and are also used as biological agents [29,33,37]. Migratory monarch populations have been found to have fewer viruses than stationary populations; therefore, reduced migration increases the risk of infection [38].

Some research has focused on the protozoan Neogregarine parasite *Ophryocystis elektroscirrha* (OE), which infects only monarch butterflies (*Danaus plexippus*) and queen butterflies (*Danaus gilippus*) [26,27,29,39]. Butterflies may also harbor parasitoid organisms that lay their eggs on/in other insects and their developing forms, such as several parasitoid wasps and tachinid flies. Some are also used as biological agents to control caterpillars [25].

It is well known that some pathogens can be transmitted between different species of pollinators [40,41,42]. Usually, the transmission of pathogens occurs from farmed species such as the honeybee (*Apis melifera*) to wild populations [41,43,44,45,46]. The two main modes of transmission are spillover (parasites originate in managed insects and spread to wild species) and spillback (parasites originate in wild insects, spread to managed species where they reach high prevalence, and spread back from managed to wild species) [45]. Facilitation (parasites originate in wild insects, competition for resources with managed species causes stress to wild species) may also lead to a decline in wild pollinator species due to the high density of managed bees and the increased competition for adequate food [45,47]. Visiting flowers to collect nectar and pollen allows interspecific transmission or at least the vectoring (without infection) of beneficial or pathogenic microbes between different pollinator groups [42,48]. The deposition of pathogens on flowers and their survival until the arrival of a new host varies among plant species, possibly due to the attractiveness or interaction of pollinators with flowers [49,50]. In recent studies, at least one bee pathogen (trypanosomes, neogregarines, and/or *Nosema* spp.) was detected in more than 70% of flower species from different areas, and the prevalence of the pathogen varied greatly among these plants [51,52]. The virus type and the flower type have an influence on the distribution of viruses on plants [53].

Several pathogenic viruses such as deformed wing virus (DWV), acute bee paralysis virus (ABPV), black queen cell virus (BQCV), Sacbrood bee virus (SBV), and Lake Sinai virus 3 (LSV3) infect honeybees. Some previous studies have confirmed the presence of these honeybee viruses in other insects, but only among members of the Apidae family [41,54,55,56,57,58]. Slovenia is a hotspot of butterfly and moth diversity, with over 3600 species estimated to be present in the country [59] (pp. 20–21). On the other hand, Slovenia is a country with a very long beekeeping tradition and an extremely high density of apiaries and bee colonies. On average, there are more than 10 bee colonies per square kilometer (data from the National Register of Apiaries in Slovenia for the year 2021). Therefore, the transmission of pathogenic viruses from honeybees to wild pollinators such as butterflies could be significant and contribute to the decline of affected local butterfly populations, which is another threat to those mentioned above. In addition, the question arises of whether butterflies could play a role in the spread of bee viruses and thus influence the problem of virus infections in honeybees.

The objective of this study was to determine if the most common and pathogenic honeybee viruses could be detected in other groups of wild pollinators, particularly, butterflies. Therefore, samples of 120 butterflies of different species collected from four different locations in Slovenia where honeybees are present were analyzed for the presence of six honeybee viruses. Our study aimed to explore this potential threat to butterflies in order to complement conservation measures.

## 2. Materials and Methods

### 2.1. Sampling

A total of 120 adult butterflies were sampled on flowering plants or in flight at four different locations in Slovenia between 7 and 21 July 2021. At each location, butterflies from three different families (10 butterfly specimens of each family), as well as 10 workers of the Carniolan honeybee (*Apis mellifera carnica*) were collected.

Sterile butterfly nets were used for sampling, so that only 10 butterflies of one taxon were captured at the same location with the same net. Depending on species abundance, butterflies of the families Pieridae, Nymphalidae, and Hesperiidae were sampled at locations #1 and #2, and butterflies of the families Pieridae, Nymphalidae, and Lycenidae were sampled at locations #3 and #4 (Table 1). All samples were frozen and stored at −60 °C until use.

### 2.2. Laboratory Investigations

In the laboratory, 10 samples of each taxon collected from the same location were pooled into a single sample containing a single species, genus, or family for further study. The pooled butterfly samples of the same taxon were treated as one sample for further testing. Each sample was homogenized in the laboratory with 5 mL of RPMI-1640 medium (Gibco, UK). The suspension was centrifuged at 2500× *g* for 5 min. Total RNA was isolated from each sample using the QIAamp viral RNA mini kit (Qiagen, Germany) according to the manufacturer’s instructions.

The RNA of six honeybee viruses—ABPV, BQCV, CBPV, DWV-A, SBV, and LSV3—was detected by specific quantitative real-time reverse transcription and polymerase chain reaction (RT-qPCR) using primers and probes previously described [60]. Briefly, the RT-qPCR assay was performed in a single step using the QuantiNova Pathogen +IC Kit (Qiagen, Hilden, Germany) and run for each honeybee virus separately. The RT-qPCR mix consisted of 5 µL of QuantiNova Master Mix, 2 µL of 10× Internal Control (IC) Probe Assay, 1 µL of IC (1:100), 4.5 µL of deionized water, 1 µL of forward primer (200 nM), 1 µL of reverse primer (200 nM), 0.5 µL of probe (100 nM), and 5 µL of the extracted RNA, for a total of 20 µL as the final volume. Thermal cycling was performed on an Mx3005P thermocycler (Stratagene, La Jolla, CA, USA) in the following conditions: 20 min at 50 °C, 2 min at 95 °C, followed by 45 cycles of 15 s at 95 °C, 30 s at 60 °C, and 30 s at 60 °C. Into each run, the positive control was included, prepared as a mixed suspension of previously determined positive field samples of the six different viruses (ABPV, BQCV, CBPV, DWV-A, SBV, and LSV3). A negative control was prepared and consisted of only 160 µL of RPMI-1640 medium (Gibco, Paisley, UK). The result of individual samples was considered positive when the Ct (cycle threshold) value was <45 and negative when the Ct (cycle threshold) value was >45. For the determination of the copy number of each virus in the tested samples, the standard for each tested virus was constructed artificially using a plasmid vector with a known viral RNA copy number. Five 10-fold dilutions of the standards, ranging from 10^−3^ to 10^−7^, were prepared and added to each RT-qPCR run. The exact number of RNA viral molecules in individual samples was determined from the standard curve for each of the six honeybee viruses.

### 2.3. Data Analysis

The viral load of each butterfly and honeybee sample was quantified. Firstly, the results were expressed as the number of detected viral copies in 5 µL of extracted RNA, with the detection limit for individual viruses around 100 copies. Each result was then calculated to the log_10_ average copy number per 10 butterflies/10 honeybees (pool sample) using the following equation: average copy number = copies/5 µL extracted RNA × 35.71 (140 µL from 5 mL of butterfly/honeybee supernatant for RNA extraction) × 12 (5 µL from 60 µL of total extracted RNA for in qPCR mix) = 428.52; log_10_ (428.52) = 2.63. The determined conversion factor (2.63) was calculated according to previously published recommendations [18] and to the volumes used in the RT-qPCR assays in this study. The results for each sample were analyzed using MxPro-Mx3005P v4.10 software (Stratagene, La Jolla, CA, USA).

For better visualization, the number of viral copies in each positive sample was converted to a log10 function and used to perform the heatmap analysis.

The analysis of results was performed according to the sample and to the sampling location. Therefore, all butterfly samples with a positive quantitative real-time RT-qPCR signal were analyzed by taxon (Pieridae, Nymphalidae, Hesperiidae, and Lycenidae) and compared to the honeybee samples. For the analysis by taxon, we summed the number of all positive results for each group and converted the result to a percentage corresponding to the total possible number of all results (e.g., 4 samples from a group multiplied by 6 viruses tested gives 24 possible results for that group). The analysis by the sampling location (Ljubljana NE, Ljubljana W, Ribnica, Žabljek) was performed in the same way.

Finally, we compared the results of this study with the local density of apiaries in the sampled area. Based on the data from the National Register of apiaries, we studied the surrounding area within a radius of 3 km.

## 3. Results

The results of the quantitative real-time RT-PCR are presented as negative (no Ct value) or positive (with Ct value) for each sample. Only 4 of the 12 (33.3%) butterfly samples were completely negative for honeybee viruses. The other samples showed low viral loads compared to the honeybees, with Ct values ranging from 28.18 to 39.82 (Table S1 in Supplementary Material). Three butterfly samples (25.0%) tested positive for one of the viruses tested, three samples (25.0%) tested positive for two viruses, one sample (8.3%) tested positive for three viruses, and one sample (8.3%) tested positive for four bee viruses (Table 2).

None (0%) of the butterfly samples were positive for CBPV and DWV-A virus. Two (16.7%) butterfly samples were positive for ABPV and LSV3, five (41.7%) samples were positive for BQCV, and seven (58.3%) samples were positive for SBV.

In comparison, all honeybee samples were positive for at least one of the viruses tested. None (0%) were positive for CBPV, two (50%) samples were positive for DWV-A, and all four (100%) bee samples were positive for ABPV, BQCV, LSV3, and SBV. The Ct values of the positive samples are reported in Table 2. The number of copies of viral RNA in the samples was also determined for all positive samples (Table 2).

**Figure 1:**
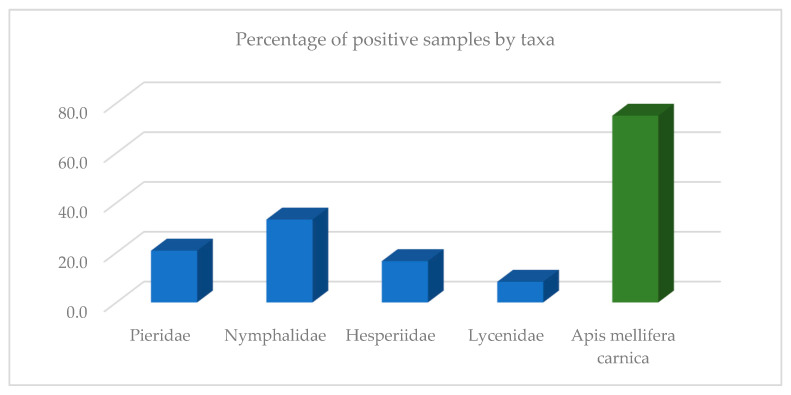
Percentage of positive samples detected in butterflies in comparison to honeybee samples.

The results of the data analysis of butterfly taxa showed that the family Nymphalidae reported the highest percentage of positive results (33.3%), followed by Pieridae (20.8%), Hesperiidae (16.6%), and Lycenidae (8.3%). For bees, 75% of the samples were positive (Figure 1).

**Figure 2:**
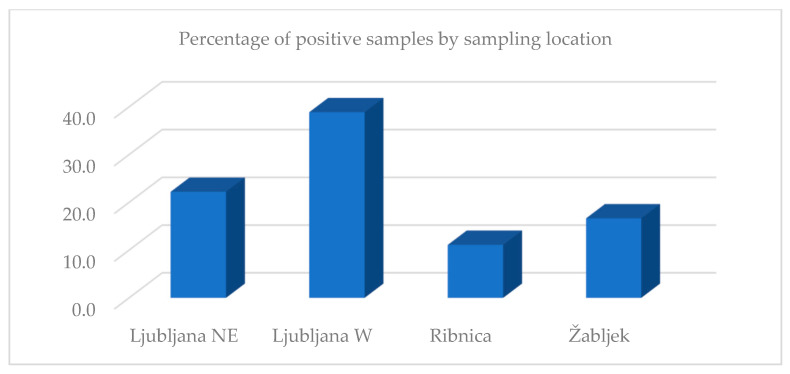
Percentage of positive butterfly samples compared by sampling location.

The analysis by sampling location showed that Ljubljana W had the highest percentage of positive results (38.9%), followed by Ljubljana NE (22.2%), Žabljek (16.7%), and Ribnica (11.1%). The bee samples by location were not analyzed because only one bee sample was obtained from each location (Figure 2).

**Figure 3:**
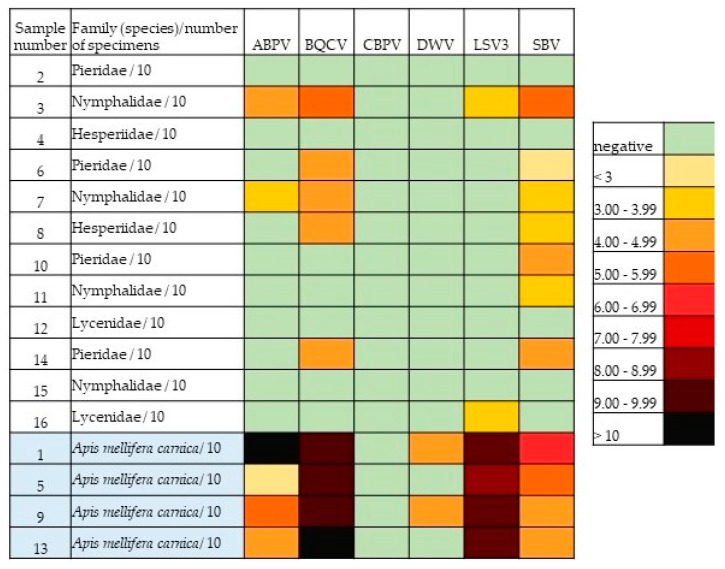
Heatmap analysis of the distribution of the detected viral load in quantitative real-time RT-PCR (number of viral copies for each positive sample converted to log_10_). Green represents a negative result. Light yellow and yellow represent weak positive results, and dark red and black represent strong positive results.

Using heat map analysis, we found a clear difference in viral load between butterfly and bee samples. The bee samples contained significantly higher virus levels compared to the butterfly samples (Figure 3).

When comparing the percentage of positive butterfly samples in each sampling location and the density of apiaries within 3 km from the sampling site, we found a higher density of apiaries in the area where the percentage of positive butterfly samples was higher. The highest number (67) of apiaries was counted around the sampling point Ljubljana W, with 38.9% of positive butterfly samples, followed by Ljubljana NE (50 apiaries, 22.2% of positive samples), Žabljek (22 apiaries, 16.7% of positive samples), and Ribnica (21 apiaries, 11.1% of positive samples). A correlation analysis was not performed due to the small sample size.

## 4. Discussion

The aim of this study was to determine the presence of pathogenic bee viruses in butterflies, as previously confirmed in bumblebees [56,58]. To our knowledge, this is the first study to demonstrate the presence of pathogenic bee viruses in butterflies. Since many butterfly species are threatened and declining, any information on the factors that could negatively affect their survival is very important for planning effective conservation measures.

To avoid taxonomic and location biases, we collected butterflies of four different families: Pieridae, Nymphalidae, Hesperiidae, and Lycenidae (Table 1) at four different locations that differed in habitat characteristics and apiary density. The bees were sampled at the same locations and times as the butterflies, primarily as evidence of an environmental burden of bee viruses at a sampling site. The presence of four bee viruses, i.e., ABPV, BQCV, LSV3, and SBV, was detected in butterflies using quantitative real-time RT-PCR, as shown in Table 2. In some samples, we found the simultaneous presence of up to four different viruses, which could be due to the combination of individual butterflies in the pooled samples of 10 specimens but also to the fact that multiple viruses were actually present in individual butterflies.

Heatmap analysis (Figure 3) was performed to obtain a better overview of the viral load by quantitative real-time RT-PCR for each sample/virus combination. Evident differences were observed between the butterfly and the bee samples. The amount of virus was significantly lower in the butterfly samples than in the positive bee samples. CBPV was not found in any of the butterfly or bee samples, which is not surprising since CBPV is one of the most pathogenic viruses in adult honeybees and is therefore unlikely to be observed in apparently healthy individuals outside of hives [61]. We also did not detect DWV-A in butterflies, but it was present in two of the four bee samples in low levels. For ABPV, BQCV, CBPV, and DWV-A, their presence in butterflies seemed to follow a similar pattern as in honeybees, but the viral loads were much lower in butterflies, so caution should be taken here; an exception was LSV3. Despite the high viral load of LSV3 in the bee samples, this virus was not detected at comparable levels in the butterflies; we do not know the reason of this discrepancy. Since LSV3 is a relatively newly discovered virus [62], not all of its characteristics are known. Of the viruses detected in butterflies, SBV was the most common (58.3%), followed by BQCV (41.7%). The high occurrence of BQCV was not surprising, as it is the most frequently detected virus in honeybees in Slovenia (83.3–100%), followed by DWV-A (4.8–70%), ABPV (3.7–79.8%), SBV (3.7–66.7%), and CBPV (0–19.1%) [60,63]. In the present study, all four bee samples were strongly positive for BQCV, indicating a high viral load on flowers at the studied locations. On the other hand, a discrepancy can be seen in regard to SBV, which was the most common virus in butterflies in this study, although it is not very common in honeybees in Slovenia [63]. However, in some previous studies, less sensitive methods (conventional RT-PCR method) were used for virus detection in honeybee samples [63]. All four bee samples in this study were also positive for SBV, but with a lower viral load compared to other viruses (Figure 3). In addition, the detected viral load of SBV was similar in the bee and butterfly samples. Perhaps, this can be explained by the fact that SBV causes a brood disease, whereas the foraging bees carry the virus only in the glands on their heads [64,65]. When a bee collects pollen and nectar on flowers, salivary particles containing this virus could contaminate the flower [66], perhaps in greater quantity than other viruses.

Comparing the viral load of the butterfly samples in relation to butterfly families (Figure 1), we found that butterflies from the family Nymphalidae were most heavily loaded with viruses, followed by Pieridae. Since the butterflies of these families (both belong to the same superfamily Papilionoidea) are larger than those from the other two families, it is possible that they can pick up and transmit more viruses on their bodies, and perhaps the family’s preference for certain flowers also plays a role. When analyzing the viral load of butterflies by sampling location, the highest load was found in butterflies in Ljubljana-NE, followed by Ljubljana-W, Žabljek, and Ribnica. The city of Ljubljana and its immediate surroundings (municipality) is an area with an extremely high density of bee colonies (on average, more than 20 colonies/square kilometer) and a high number of beekeepers and apiaries. In previous studies, this area was also found to have the highest prevalence of viral infections in honeybee colonies [60]; therefore, we hypothesize that a slightly higher environmental burden of bee viruses is present in this area.

The low number of viral RNA copies in the butterfly samples compared to the high number of positive bee samples in our study suggests a more likely passive transmission of viruses to butterflies. This is emphasized by the match between the percentage of positive butterflies and the density of apiaries at the locations studied. Therefore, butterflies may be considered as potential indicators of viral load in the environment. Because this is only the first study on bee viruses in butterflies, many questions remain unanswered, and further studies are required to clarify transmission routes and differences in viral loads among these insect groups.

## 5. Conclusions

In our study, we detected pathogenic honeybee viruses in butterflies for the first time. ABPV, BQCV, LSV3, and SBV were detected in both butterflies and honeybees collected at the same locations, while DWV-A was found only in honeybees. CBPV was not detected in either butterflies or honeybees. Our results clearly indicate that infected honeybees are a prerequisite for virus detection in cohabiting butterflies. There was not a single case where butterflies had a virus that was not also found in honeybees at the same sampling location. Therefore, there is a high probability that the direction of transmission is from honeybees to butterflies and not vice versa. The virus levels were much lower in the butterflies than in the honeybees; therefore, the likelihood of transmission of bee viruses to other organisms by butterflies is very low or not significant. We assume that the possible sites of virus transmission are the flowers of nectar plants visited by both honeybees and butterflies.

## Figures and Tables

**Table 1:** List of butterfly samples by location, habitat type, taxon, and species/genus/family. At each of the four locations, 10 visually healthy honeybees of the species *Apis mellifera carnica* were sampled on the flowering plants in the same way as the butterflies (sample numbers 1, 5, 9 and 13). Legend: Ljubljana-NE = north-eastern part of the periphery of Ljubljana (46.087191, 14.553941); Ljubljana-W = western part of the periphery of Ljubljana (46.053369, 14.467093); Ribnica = town in the southern part of Slovenia (45.718746, 14.776496); Žabljek = village in the eastern part of Slovenia (46.350380, 15.563231).

Sample Number	Location	Habitat Type	Taxon	Species/Number of Specimens
1	1/Ljubljana-NE	dry meadows, shrubs, forest path	Apidae	*Apis mellifera carnica*/10
2	1/Ljubljana-NE		Pieridae	*Leptidea* sp./10
3	1/Ljubljana-NE		Nymphalidae	*Maniola jurtina*/10
4	1/Ljubljana-NE		Hesperiidae	*Tymelicus* sp./10
5	2/Ljubljana-W	wet meadows, forest edge	Apidae	*Apis mellifera carnica*/10
6	2/Ljubljana-W		Pieridae	*Gonopteryx rhamni*/10
7	2/Ljubljana-W		Nymphalidae	*Maniola jurtina*/10
8	2/Ljubljana-W		Hesperiidae	*Ochlodes sylvanus*/9, *Tymelicus* sp./1
9	3/Ribnica	flowering edges along the railway, extensively managed meadows	Apidae	*Apis mellifera carnica*/10
10	3/Ribnica		Pieridae	*Pieris rapae*/10
11	3/Ribnica		Nymphalidae	*Melanargia galathea*/10
12	3/Ribnica		Lycenidae	*Polyommatus icarus*/10
13	4/Žabljek	cultivated and extensively managed wet meadows	Apidae	*Apis mellifera carnica*/10
14	4/Žabljek		Pieridae	*Pieris* sp. (*P. napi*/1; *P. brassicae*/1; *P. rapae*/8)
15	4/Žabljek		Nymphalidae	*Coenonympha pamphilus*/10
16	4/Žabljek		Lycenidae	*Polyommatus icarus*/10

**Table 2:** Viral RNA copy number in each tested sample, which consisted of 10 butterflies/10 honeybees for each virus individually.

		ABPV	BQCV	CBPV	DWV	LSV3	SBV
Sample Number	Family (Species)/Number of Specimens	No. of Copies	No. of Copies	No. of Copies	No. of Copies	No. of Copies	No. of Copies
2	Pieridae/10	0	0	0	0	0	0
3	Nymphalidae/10	9.5 × 10^4^	4.7 × 10^5^	0	0	9.8 × 10^3^	1.1 × 10^5^
4	Hesperiidae/10	0	0	0	0	0	0
6	Pieridae/10	0	2.1 × 10^4^	0	0	0	8.5 × 10^2^
7	Nymphalidae/10	5.5 × 10^3^	5.6 × 10^4^	0	0	0	4.7× 10^3^
8	Hesperiidae/10	0	4.4 × 10^4^	0	0	0	4.7 × 10^3^
10	Pieridae/10	0	0	0	0	0	1.1 × 10^4^
11	Nymphalidae/10	0	0	0	0	0	1.2 × 10^3^
12	Lycenidae/10	0	0	0	0	0	0
14	Pieridae/10	0	2.5 × 10^4^	0	0	0	3.9 × 10^4^
15	Nymphalidae/10	0	0	0	0	0	0
16	Lycenidae/10	0	0	0	0	1.7 × 10^3^	0
1	*Apis mellifera carnica*/10	5.4 × 10^12^	5.0 × 10^9^	0	1.2 × 10^4^	6.6 × 10^9^	3.3 × 10^6^
5	*Apis mellifera carnica*/10	8.5 × 10^2^	1.5 × 10^9^	0	0	4.7 × 10^8^	1.7 × 10^5^
9	*Apis mellifera carnica*/10	1.7 × 10^5^	3.7 × 10^9^	0	1.0 × 10^4^	1.5 × 10^9^	6.8 × 10^4^
13	*Apis mellifera carnica*/10	2.4 × 10^4^	5.1 × 10^10^	0	0	3.6 × 10^9^	3.2 × 10^4^

## Data Availability

The data presented in this study are available on request from the corresponding author.

## Supplementary Materials

The following is available online at https://www.mdpi.com/article/10.3390/insects13100925/s1, Table S1: Obtained Ct values from quantitative real-time RT-PCR for tested butterfly and honeybee samples for each virus.
