# Supplementary material for: First Detection of Honeybee Pathogenic Viruses in Butterflies
Source: Insects. 2022 Oct 12;13(10):925. doi: 10.3390/insects13100925 (PMC9604290; doi:10.3390/insects13100925)
Supplement: Supplementary file 1 [file insects-13-00925-s001.zip › insects-1908131-supplementary.pdf]

Table S1. Obtained Ct values from quantitative real-time RT-PCR for tested butterfly and honeybee samples for each virus.

		**ABPV**	**BQCV**	**CBPV**	**DWV**	**LSV3**	**SBV**
Sample number	Family (species) / number of specimens	Ct value	Ct value	Ct value	Ct value	Ct value	Ct value
2	Pieridae / 10	no Ct	no Ct	no Ct	no Ct	no Ct	no Ct
3	Nymphalidae / 10	**35.37**	**28.18**	no Ct	no Ct	**37.08**	**31.55**
4	Hesperiidae / 10	no Ct	no Ct	no Ct	no Ct	no Ct	no Ct
6	Pieridae / 10	no Ct	**36.62**	no Ct	no Ct	no Ct	**38.97**
7	Nymphalidae / 10	**38.33**	**30.53**	no Ct	no Ct	no Ct	**36.2**
8	Hesperiidae / 10	no Ct	**30.79**	no Ct	no Ct	no Ct	**36.12**
10	Pieridae / 10	no Ct	no Ct	no Ct	no Ct	no Ct	**34.86**
11	Nymphalidae / 10	no Ct	no Ct	no Ct	no Ct	no Ct	**38.06**
12	Lycenidae / 10	no Ct	no Ct	no Ct	no Ct	no Ct	no Ct
14	Pieridae / 10	no Ct	**30.42**	no Ct	no Ct	no Ct	**39.82**
15	Nymphalidae / 10	no Ct	no Ct	no Ct	no Ct	no Ct	no Ct
16	Lycenidae / 10	no Ct	no Ct	no Ct	no Ct	**39.75**	no Ct
1	*Apis mellifera carnica* / 10	**16.41**	**18.03**	no Ct	**36.3**	**15.77**	**26.68**
5	*Apis mellifera carnica* / 10	**40.14**	**19.3**	no Ct	no Ct	**19.96**	**30.94**
9	*Apis mellifera carnica* / 10	**34.71**	**18.35**	no Ct	**36.52**	**18.05**	**32.33**
13	*Apis mellifera carnica* / 10	**36.8**	**15.48**	no Ct	no Ct	**16.74**	**40.12**
